# Uterine Inflammation Changes the Expression of Cholinergic Neurotransmitters and Decreases the Population of AChE-Positive, Uterus-Innervating Neurons in the Paracervical Ganglion of Sexually Mature Gilts

**DOI:** 10.3390/ani12131676

**Published:** 2022-06-29

**Authors:** Bartosz Miciński, Barbara Jana, Jarosław Całka

**Affiliations:** 1 Department of Clinical Physiology, Faculty of Veterinary Medicine, University of Warmia and Mazury, Oczapowskiego 14, 11-041 Olsztyn, Poland; calkaj@uwm.edu.pl; 2 Division of Reproductive Biology, Institute of Animal Reproduction and Food Research of the Polish Academy of Sciences, Tuwima 10, 10-748 Olsztyn, Poland

**Keywords:** endometritis, cholinergic innervation, paracervical ganglion, uterine neurons, chemical coding, immunocytochemistry, pig

## Abstract

**Simple Summary:**

Endometritis, both with non-infectious and infectious backgrounds, is one of the most prevalent pathological states among domestic animals. In animals, it generates severe economic problems, including lowered reproductive indices and rising medical treatment costs, and in women, it might lead to severe fertility impairment. In order to determine how the autonomic nervous system responds to such a pathological state, an experimental group of pigs were treated with Escherichia coli injection into the uterine horns, and several ganglions responsible for innervation of this organ were examined, including the paracervical ganglion located on both sides of the broad ligament of the uterus. The results clearly showed a strong impact of the inflammation on the chemical coding of neurons, some even synthesizing neurotransmitters de novo such as the GAL-expressing perikarya. Additionally, applied injections decreased the number of parasympathetic, acetylcholinesterase-expressing neurons implying the importance of the cholinergic population to keep the inflammation under control. The obtained data serve as a basis for the future implementation of modern treatment and enhancements in animal breeding.

**Abstract:**

The focus of this study was based on examining the impact of endometritis on the chemical coding of the paracervical ganglion (PCG) perikaryal populations supplying pig uterus. Four weeks after the injection of Fast Blue retrograde tracer into uterine horns, either the *Escherichia coli* (*E*. *coli*) suspension or saline solution was applied to both horns. Laparotomy treatment was performed for the control group. Uterine cervices containing PCG were extracted on the eighth day after previous treatments. Subsequent macroscopic and histopathologic examinations acknowledged the severe form of acute endometritis in the *E*. *coli*-treated gilts, whereas double-labeling immunofluorescence procedures allowed changes to be analyzed in the PCG perikaryal populations coded with vesicular acetylcholine transporter (VAChT) and/or somatostatin (SOM), vasoactive intestinal polypeptide (VIP), a neuronal isoform of nitric oxide synthase (nNOS), galanin (GAL). The acetylcholinesterase (AChE) detection method was used to check for the presence and changes in the expression of this enzyme and further confirm the presence of cholinergic perikarya in PCG. Treatment with *E*. *coli* resulted in an increase in VAChT+/VIP+, VAChT+/VIP−, VAChT+/SOM+, VAChT+/SOM−, VAChT+/GAL− and VAChT+/nNOS− PCG uterine perikarya. An additional increase was noted in the non-cholinergic VIP-, SOM- and nNOS-immunopositive populations, as well as a decrease in the number of cholinergic nNOS-positive perikarya. Moreover, the population of cholinergic GAL-expressing perikarya that appeared in the E. coli-injected gilts and *E*. *coli* injections lowered the number of AChE-positive perikarya. The neurochemical characteristics of the cholinergic uterine perikarya of the PCG were altered and influenced by the pathological state (inflammation of the uterus). These results may indicate the additional influence of such a state on the functioning of this organ.

## 1. Introduction

A female-specific, ganglionic cluster located on both sides of the ligamentum latum uteri, more precisely at the uterovaginal junction, is known as the paracervical ganglion (PCG). The described ganglion constitutes a part of a larger pelvic plexus. It consists of both a sympathetic and parasympathetic component, supplying the reproductive system and urinary tract organs [1,2,3] with both types of nerve fibers. Moreover, a small population of non-adrenergic, non-cholinergic (NANC) neurons has been described [4,5]. Its cholinergic neurons are responsible for a variety of parasympathetic actions, such as dilatation of uterine arteries [6]. Earlier studies using Fast Blue (FB) fluorescent neuronal retrograde tracer confirmed that the pig uterus is innervated by terminals originating from many autonomic and sensory ganglia, including PCG [3,7].

Metritis/endometritis developing in response to non-infectious, as well as infectious bacteriological factors, is one of the most prevalent pathological states among domestic animals that alters neurotransmitter secretion in autonomic ganglia such as the above-mentioned PCG. Moreover, although mainly appearing after parturition, it might occur after insemination and natural mating [8,9,10]. It is characterized by generating severe economic troubles, including lowered reproductive indices and rising medical treatment costs. To demonstrate the presence of AChE activity, affirm the cholinergic phenotype of VAChT-positive neurons, and confirm and help determine the impact of endometritis on neuronal neurotransmitter secretion, a modified Karnovsky–Roots method [11] may be used. Acetylcholinesterase (AChE) is present in the neuromuscular junctions and brain cholinergic synapses, as well as other tissues, and plays a pivotal role in neurotransmission acting by degradation of acetylcholine (ACh). It is a commonly used indicator for cholinergic function in brain tissues [12,13]. There are not any known contraindications to the staining of neuronal bodies; however, according to one concept, AChE-staining should not be used as a reliable marker for cholinergic nerves, as it has been shown to stain sympathetic nerve fibers as well [14,15,16]. Other authors report no problems with the uterus tissue [17].

Past reports showed the existence of uterus-supplying perikarya inside the pig PCG [7]. Studies focusing on double immunofluorescence staining of PCG neurons in rodents reported populations expressing both choline acetyltransferase (ChAT) and vesicular ACh transporter (VAChT) most often used as cholinergic neurons markers [1,18]. Moreover, other authors confirmed the coexistence of various substances in these types of neurons. The discovered substances include substance P (SP), vasoactive intestinal polypeptide (VIP), galanin (GAL), a neuronal isoform of NO synthase (nNOS), somatostatin (SOM), and neuropeptide Y (NPY) [3,19,20,21,22,23,24]. There are insufficient data on the chemical coding of uterine-supplying perikaryal populations in the porcine PCG [3,7]. Interestingly, few data exist on changes in the expression of mentioned substances in cholinergic uterine neurons in PCG for a state such as endometritis.

Studies on rats show that uterine inflammation causes behavior changes, probably in response to visceral pain [25]. Moreover, a rise in the number of SP-positive neurons for sensory dorsal root ganglia (DRG) was recorded [26]. An earlier report showed that bacteria-induced inflammation caused a decrease in the nerve fiber population in the pig uterus, including nerve terminals of the noradrenergic type [27]. Papers concerning the consequences of uterine inflammation on sensory ganglia [28], as well as the caudal mesenteric ganglion (CaMG) [29], showed decreasing numbers of uterine supplying perikarya in both. A recent paper presented a severe decrease in the number of uterine perikarya in the PCG of *Escherichia coli* (*E*. *coli*)-treated gilts, as well as an impact of endometritis on chemical coding of the sympathetic neurons [30]. Thus, these facts combined allow us to form the hypothesis that uterine inflammation has a detectable impact on the parasympathetic, uterus-supplying neuronal cells in PCG of sexually mature gilts and alters the expression of various neurotransmitters. For a more in-depth understanding of etiopathogenesis, it is essential to investigate what changes occur in the cholinergic innervation of the inflamed uteri. Since it may be significant for the course and/or outcome of this pathological state, the results may be considered important in the potential improvement of survivability, breeding indicators for animals, and profitability for breeders. The use of a porcine model in any type of biomedical research, including studies on the reproductive system, has already been well-grounded. This type of study is possible due to the essential similarity and comparability to humans [31,32]. In such an aspect, the study may be significant for women suffering from uterus inflammation. These results may be treated as a basis for the introduction of modern, therapeutic measures beneficial to humans as well.

The objective of the following study was to determine the number of uterine perikarya expressing VAChT and/or VIP, GAL, SOM, nNOS, and AChE in the PCG after *E. coli*-evoked uterine inflammation in gilts.

## 2. Materials and Methods

### 2.1. Animals

The research material consisted of 11 (n = 11) sexually mature, crossbred gilts showing signs of behavioral estrus, which was confirmed with the use of a tester boar. All gilts weighed from 90 to 120 kgs and were 7–8 months of age; they were separated into either *E*. *coli* (n = 4), saline (n = 3), or control (n = 4) group. The conditions in which they were kept were thoroughly described in a previous article [30]. It is essential to note that all parameters were set in accordance with the instructions and agreement of the Local Ethical Committee in Olsztyn, Poland, and the authors used all measures necessary to keep the stress reaction to surgery and the post-surgical period at a minimum level, in accordance with Consent no. 65/2015.

### 2.2. Experimental Procedures

All of the necessary doses, drug trade names, and details for premedication, general anesthesia, surgical treatment, FB, and *E*. *coli* injection procedures in particular groups, have already been described in one of the authors’ previous papers [30]. The procedures are briefly described in what follows.

Premedication administered to the examined gilts on day 0 of the described experiment, which was day 17 of the first studied estrous cycle, consisted of azaperone, injected intramuscularly atropine injected intramuscularly, as well as ketamine hydrochloride, injected intravenously with which general anesthesia was induced and sustained with reapplication after every five minutes and injected intravenously.

An abdominal incision was carried out to expose both uterine horns and inject an aqua solution of FB retrograde fluorescent neuronal tracer. The tracer was thoroughly and evenly spread into the walls of paraoviductal, middle, and paracervical parts of both left and right horns with the use of a Hamilton syringe. In total, 13 separate FB injections were performed in each area. Standard procedures such as a 60 s stationary needle time, as well as gauze rinsing and wiping, were also carried out. Uterus-innervating neurons were visualized via FB. The time to reach innervation sources for this tracer is four weeks.

Gilts were subjected to another anesthesia on day 28, which was the expected third day of the third studied estrous cycle (marking the start of the remaining procedures). The procedures followed were identical to the ones described earlier. A laparotomy allowed applying an *E*. *coli* suspension and saline solution to the experimental and saline groups. The control group was subjected to laparotomy treatment only. On day 11 of the third studied estrous cycle (marking the period of 8 days after the laparotomies), euthanasia was carried out on all the animals. For this task, an overdose of ketamine hydrochloride injected intravenously was used. Subsequently, an infusion of a 4% buffered paraformaldehyde through the pars ascendens aortae was carried out. Collection of all the ganglia, including PCG together with the uterine cervix, broad ligament of the uterus, and urinary bladder as orientation guides were performed following the finished paraformaldehyde infusion. Immersive tissue postfixation was carried out immediately after the last step, with a subsequent rinsing using the phosphate buffer performed over the next two days. Lastly, all prepared tissues were stored at 4 °C immersed in a buffered sucrose solution.

Correctly trimmed and prepared tissues containing potential PCG were frozen and kept at −80 °C until further immunohistochemical proceedings. The methods of the precise establishment of the inflammation form, along with the results, were similarly described in the authors’ earlier articles [31,33].

### 2.3. Immunohistochemical Analysis

Tissues were cut into 14 µm sections and subsequently mounted onto the slides coated earlier with chrome alum. The following examination, using a Zeiss AxioImager (Zeiss, Oberkochen, Germany) microscope containing a fluorescent module (V1 module) and appropriate filters (330–385 nm excitation filter and 420 nm barrier filter), of the examined slices allowed us to subject the FB neuron-containing sections to immunohistochemical double-labeling procedures. All of the steps, as well as the required reagents for this method, were thoroughly described in our last article [30]. As this study focuses on the parasympathetic part of the examined ganglion, antibody combinations are the only differing part (Table 1). Standard controls, i.e., pre-absorption for the neuropeptide antisera with appropriate antigen (20 μg of antigen/mL diluted antiserum) and the omission, as well as the replacement of all primary antisera by non-immune sera, were performed to test immunohistochemical labeling. There was no fluorescence observed in any of these control stainings.

With staining procedures completed, FB-labeled perikarya were passed for further inspection under the fluorescent microscope in order to count the number and analyze the exposed antibody combinations. Photographs were taken using Zeiss AxioImager.M2′s Axiocam 705 digital monochromatic camera. All retrograde-marked neurons expressing either VAChT, SOM, VIP, GAL, or nNOS were counted in every 4th section of the paracervical ganglion. The threshold of the statistically significant differences was set at *p* < 0.05.

### 2.4. Histochemical Analysis

A modified Karnovsky–Roots method was used to present the activity or lack of AChE in the cholinergic uterus-innervating neurons. Before histochemical procedures, sections with VAChT-immunoreactive perikarya were photographed with a digital monochromatic camera (Zeiss AxioImager, Oberkochen, Germany) connected to a PC, and the coordinates of each photograph were saved in order to locate the same neuron after staining using the aforementioned method.

Sections were then incubated with a clear, green, stable medium consisting of 5 mg of acetylthiocholine iodide dissolved in 6.5 mL 0.1 M sodium hydrogen maleate buffer of pH 6.0, 0.5 mL of 0.1 M sodium citrate, 1 mL of 30 mM CuSO_4_, 1 ml water, and 1 mL of 5 mM potassium ferricyanide. Other types of cholinesterase activity were inhibited by 0.05 mM tetraisopropyl pyrophosphoramide (iso-OMPA, Sigma, Darmstadt, Germany). The incubation time was 2 h at 37 °C. Sections were then rinsed with gentle agitation for 10 min in distilled water.

After staining, VAChT-IR neurons were further checked under a microscope to count AChE-positive cells and then photographed. AChE-positive perikarya were counted for approximately 50 VAChT positive, uterine-supplying neurons for PCG of each pig.

### 2.5. Statistical Analysis

The results were acquired from a PCG examination in all three groups of this experiment and averaged per neuron with particular coding for each group. The data are expressed as percentages of the total population of uterine-supplying nerve cells stained for two substances in each group and accepted as 100%. For AChE staining, the data are expressed as a number of positively stained neurons. A one-way analysis of variance (ANOVA) was carried out using Statistica 13 software (StatSoft Inc., Tulsa, OK, USA) to determine the standard error of the mean (±SEM), followed by the Bonferroni test to validate whether the differences were statistically significant. The threshold was set at *p* < 0.05.

## 3. Results

### 3.1. The Numbers of Uterine-Supplying Neurons Containing VAChT, SOM, VIP, GAL, and nNOS in the PCG

**Figure 1:**
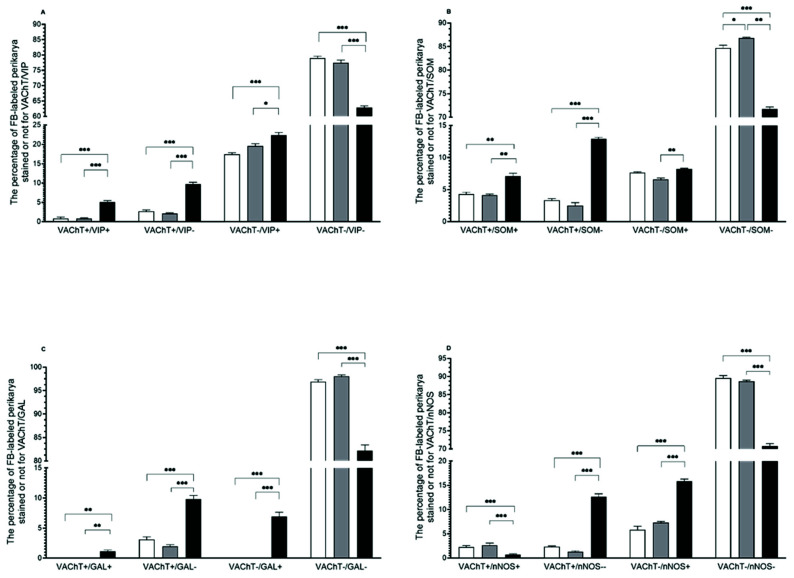
The populations (expressed as percentages, mean ± SEM) of uterine perikarya expressing vesicular acetylcholine transporter (VAChT) and/or vasoactive intestinal polypeptide (VIP) (**A**), VAChT and/or somatostatin (SOM) (**B**), VAChT and/or galanin (GAL) (**C**), and VAChT and/or neuronal isoform of nitric oxide synthase (nNOS) (**D**), as well as those without these substances in the PCG of gilts from the control (white bars), saline (grey bars), and *E*. *coli* (black bars) groups. Data are expressed as percentages of the total population of uterine perikarya stained for two substances in each group, accepted as 100%. * *p* < 0.05, ** *p* < 0.01, and *** *p* < 0.001 show differences between all groups for the same population of uterine perikarya.

**Figure 2:**
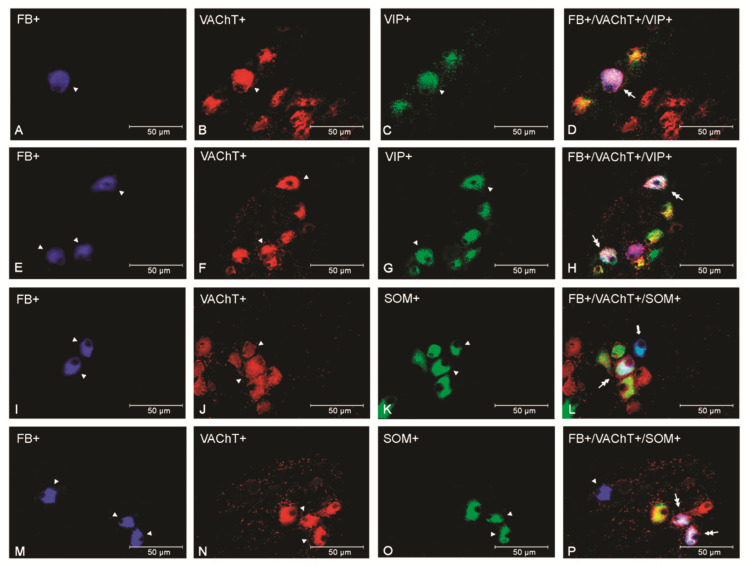
Micrographs demonstrating the presence of VAChT (**B**,**F**,**J**,**N**), VIP (**C**,**G**), and SOM (**K**,**O**) in the PCG uterine perikarya of gilts from the saline (**A**–**D**), control (**I**–**L**), and *E*. *coli* (**E**–**H**,**M**–**P**) groups. The arrowhead indicates a Fast Blue (FB)-positive neuron, a perikaryon immunoreactive to VAChT, VIP, and a perikaryon immunoreactive to SOM. The double arrow indicates an FB-positive uterine neuron expressing VAChT and VIP and VAChT and SOM. The arrow indicates an FB-positive perikaryon expressing SOM. The photographs (**D**,**H**,**L**,**P**) were made using the digital superimposition of three color channels: FB-positive (blue), VAChT-positive (red), and SOM- or VIP-positive (green). One VAChT and VIP immunoreactive uterine neuron is visible in the gilt of the saline group (**A**–**D**). In the PCG of the *E*. *coli* group, an elevated number of perikarya expressing these substances are visible (**E**–**H**). One perikaryon expressing SOM and VAChT is present in the ganglion of the control group (**I**–**L**). In the *E*. *coli* group, two perikarya expressing both of these substances are observed in the PCG (**M**–**P**).

In comparison to both the control and saline groups, the numbers of VAChT+/VIP+ and VAChT+/VIP− uterine perikarya statistically significantly increased in the PCG of the bacteria-treated gilts (*p* < 0.001). VAChT-/VIP+ population increased in control (*p* < 0.001) and saline (*p* < 0.05) groups as well (Figure 1A and Figure 2A–H), whereas the VAChT-/VIP- population size was diminished (*p* < 0.001) in relation to the saline and control groups (Figure 1).

In the PCG of *E*. *coli*-injected gilts, the number of VAChT+/SOM+ neurons was higher than that of the control and saline (*p* < 0.01) groups (Figure 1B and Figure 2I–P), whereas the population of VAChT-/SOM+ reactive perikarya noted an increase (*p* < 0.05) in comparison to the saline group only. Furthermore, a rise (*p* < 0.001) was noted in the bacterial group’ VAChT+/SOM− coded neurons when compared with other groups, whereas a decrease was present in the VAChT-/SOM- population in relation to the control (*p* < 0.001) and saline groups (*p* < 0.01) (Figure 1B). A statistically significant difference was noted between the saline and control groups only in VAChT-/SOM- coded neuronal cells (*p* < 0.05).

**Figure 3:**
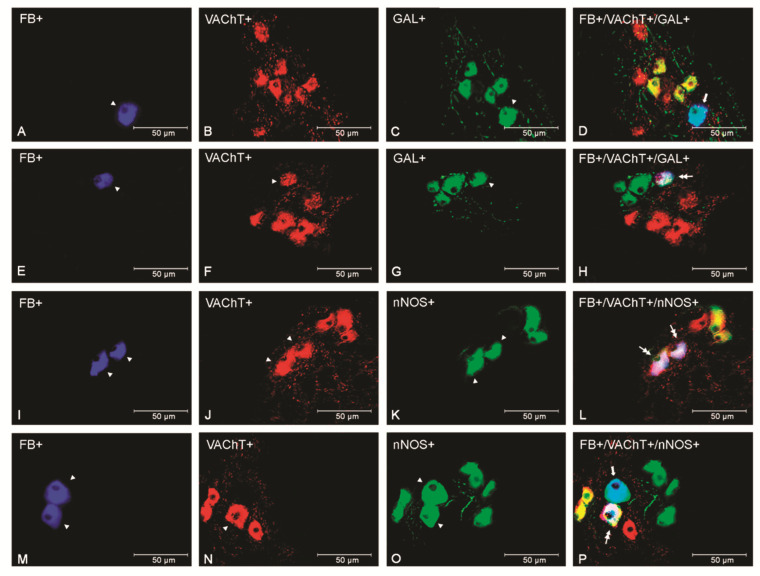
Micrographs demonstrating the presence of VAChT (**B**,**F**,**J**,**N**), GAL (**C**,**G**), and nNOS (**K**,**O**) in the PCG uterine perikarya of gilts from the control (**A**–**D**), saline (**I**–**L**), and *E*. *coli* (**E**–**H**,**M**–**P**) groups. The arrowhead indicates a Fast Blue (FB)-positive neuron, a perikaryon immunoreactive to VAChT, a neuron immunoreactive to GAL, as well as an nNOS-immunoreactive perikaryon. The double arrow indicates an FB-positive uterine neuron expressing VAChT and GAL/nNOS, whereas the arrow shows an FB-positive and GAL/nNOS immunoreactive neuron. The photographs were made using the digital superimposition of three color channels: FB-positive (blue), VAChT-positive (red), and nNOS- or GAL-positive (green). One perikaryon expressing GAL but not VAChT is present in the ganglion of the control group (**A**–**D**). In the *E*. *coli* group, a perikaryon expressing these substances is observed in the PCG (**E**–**H**). Two VAChT and nNOS immunoreactive uterine neurons are visible in the gilt of the saline group (**I**–**L**). In the PCG of the *E*. *coli* group, a decreased number of perikarya expressing these substances are visible (**M**–**P**).

*E*. *coli* treatment led to an increase (*p* < 0.01) in the population of the VAChT+/GAL− when compared with the other two groups (Figure 1C). Moreover, populations of VAChT+/GAL+ and VAChT-/GAL+ immunoreactive uterus-innervating neurons appeared (Figure 1C and Figure 3A–H) in the bacteria-treated group in relation to two other groups (*p* < 0.01 and *p* < 0.001, respectively). Additionally, the number of VAChT-/GAL- expressing population decreased (*p* < 0.001) in relation to those of both the control and saline groups.

Uterine inflammation also led to a decrease (*p* < 0.001) in the number of VAChT+/nNOS+ neurons (Figure 1D and Figure 3I–P) and an increase (*p* < 0.001) in VAChT+/nNOS− and VAChT-/nNOS+ populations in relation to the control and saline groups. Endometritis evoked a decrease (*p* < 0.001) in the number of VAChT-/nNOS- neurons in comparison to other groups (Figure 1D).

Figure 1 presents VAChT- and/or VIP-, SOM-, GAL-, and nNOS-positive uterine-innervating neurons, as well as those not showing the expression of any of these substances in the PCG in any of the three examined groups of gilts.

### 3.2. The Number of Uterine Perikarya Containing AChE in the PCG

**Figure 4:**
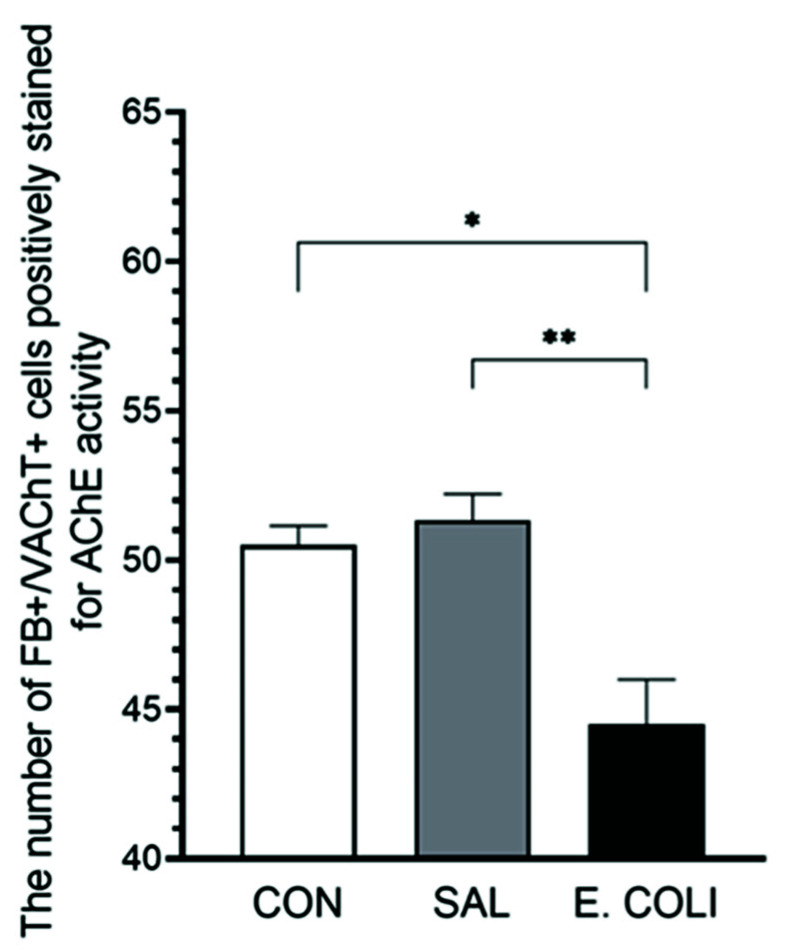
The numbers of both FB- and VAChT-positive neurons positively stained for AChE (mean ± SEM) in the PCG counted in the gilts from control (CON), saline (SAL), and *E*. *coli* (*E*. *coli*) groups (* *p* < 0.05 and ** *p* < 0.01 show the differences between groups).

**Figure 5:**
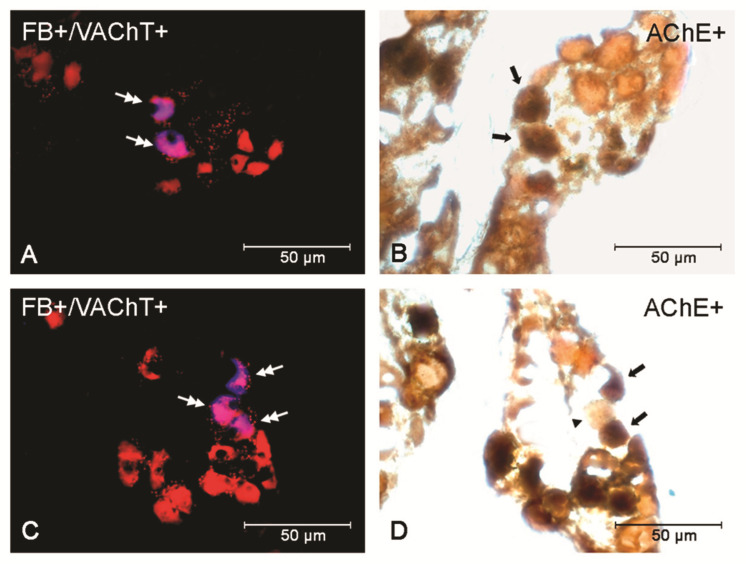
Micrographs demonstrating the presence of uterine, VAChT-positive (**A**,**C**), and AChE (**B**,**D**) expressing perikarya in the PCG of gilts from the saline (**A**,**B**) and *E*. *coli* (**C**,**D**) groups. The double arrow indicates an FB-positive neuron expressing VAChT. The arrow indicates an AChE-positive, whereas the arrowhead indicates an AChE-negative perikaryon. Two uterine neurons expressing VAChT and AChE are visible in the gilt of the saline group (**A**,**B**). A decreased number of perikarya expressing AChE are present in the PCG of the bacteria-treated group (**C**,**D**).

A decrease in the number of stained AChE-positive perikarya was noted in the *E*. *coli*-administered group in relation to the control (*p* < 0.05) and saline (*p* < 0.01) groups (44.50 ± 1.5 vs. 50.50 ± 0.65, 51.33 ± 0.88, respectively) (Figure 4 and Figure 5A–D). In the control and saline groups, all of the VAChT-positive neurons were strongly AChE-stained (thus AChE-expressing), whereas such intensive staining was not present in the *E*. *coli* group.

## 4. Discussion

The current study, for the first time, indicated changes in the expression of neurotransmitters and AChE in cholinergic uterine perikarya in the PCG of sexually mature gilts in response to uterus inflammation. *E*. *coli* suspension was applied when gilts were in the early luteal phase of the estrous cycle. Such a phase is defined by a rising level of progesterone, which has immunosuppressive characteristics and promotes inflammation development. Additionally, levels of immunostimulating LTs, 17β-estradiol (E2), and uterine PGF2α are significantly decreased throughout this phase, further aiding the development of disease [33,34]. A result was the manifestation of a severe form of acute inflammation. Inoculation of the same, as well as a lower quantity of *E*. *coli*, led to a similar situation in previous studies [35,36]. Essentially, the aforementioned form is diagnosed in the presence of a highly increased number of neutrophils. Additionally, damage to the luminal epithelium and/or gland structures is present [1]. An earlier study confirmed the incidence of this form of inflammation in the examined gilts with the use of histopathological procedures [28].

Neuronal populations showing immunoreactivity to substances studied in this article did not present statistically significant alterations in response to saline inoculation, excluding only the non-cholinergic, somatostatin-negative population. Such an occurrence indicates that surgical procedures and saline injections had no effect on the chemical coding of the examined neurons.

As mentioned in the Results section, in the bacteria-treated group, many statistically significant alterations in chemical coding were observed, with both increases and decreases in the number of specific neuronal populations. Earlier research, focused on the examination of neurotransmitter expression in all PCGs neurons in immature pigs, proved the existence of numerous cholinergic perikarya [23]. Subsequent studies performed on adult gilts confirmed changes in VAChT-immunoreactive populations of ovarian neurons in response to several factors. Similar to the current results, increased levels of VAChT-/SOM+ and VAChT-/VIP+ were noted in response to testosterone treatment, whereas decreases in VAChT-positive but VIP-negative, VAChT-immunoreactive, VIP-, nNOS-, and SOM-immunoreactive were recorded as a result of long-term E2 and testosterone administration [37,38]. Interestingly, upregulation of cholinergic, VAChT+/ChAT+ urinary bladder-innervating perikarya, as well as nNOS-, GAL-, and VIP-positive neurons, was presented in the anterior pelvic ganglion (APG; the male equivalent of PCG) in response to one-sided axotomy [39]. In contrast, in response to the same trauma, PCG uterine neurons presented no changes in VAChT or ChAT expression [40]. Both studies were, however, performed on immature animals. Moreover, resiniferatoxin and tetrodotoxin induced a drop in cholinergic, nNOS-immunoreactive urinary-bladder-supplying neuronal cells in PCG with an additional increase in non-cholinergic, nNOS-positive perikarya, similar to the current findings [24]. Studies on ER-impacting Bisphenol A confirmed a measurable impact on the number of uterine nerve fibers, in which it significantly increased the population of nitrergic nerves in either low or high doses [41].

To date, AChE activity has been identified in the nerve fibers of pigs’ ovaries, oviducts, uterine horns, and vagina [42]. Interestingly, the current results showed a statistically significant decrease in strongly stained AChE-expressing, VAChT+ neurons in the *E*. *coli* group. Although the direct mechanism is not known, it may be hypothesized that, in order to consolidate the demand for ACh and its properties and maximize its concentration in the nerve terminals, cholinergic neurons respond with a limitation of the AChE expression, as the examined enzyme is responsible for degradation of the acetylcholine neurotransmitter, thus bringing an end to cholinergic neurotransmission [43]. Moreover, it has been previously proven that AChE activity changes in ovaries in different phases of the estrous cycle, when steroid hormone levels fluctuate [44]. During the luteal phase, the immunosuppressive progesterone level rises, and the immunostimulating E2 and PGF2α levels decrease, as mentioned earlier in the Discussion section. Previous studies have shown that bacterial injections into uterine horns of gilts subsequently lowered P4 concentration [45]. Other studies have shown that E2 and P4 injections resulted in increased AChE activity in ovaries, oviducts, and horns, whereas they caused a decrease in enzyme activity in the cervix E2 [42]. It was also reported that the luteal phase length might elongate in cases of chronic uterine infections in the mare, often combined with a pyometra, due to insufficient PGF2α production necessary for luteolysis [46]. Thus, the aforementioned P4 and E2 concentration differences appearing in the course of the inflamed uterus may be treated as part of the discussed mechanism leading to a decreased AChE expression.

In physiological conditions, the plasticity of the nervous system supplying the reproductive tract is associated with changes in uterine innervation density occurring during the course of the estrous cycle and pregnancy [47,48,49,50,51,52], whereas the earlier mentioned fluctuations in various neurotransmitters of either cholinergic or non-cholinergic perikarya, directly indicate the intensified response of the autonomic nervous system to inflammation and may indicate the disrupted variety of cholinergic and non-cholinergic mechanisms. An upregulation of populations expressing VIP and other neuronal factors confirms the properties of these substances. Best recognized for its anti-inflammatory functions, VIP plays a role in the regulation of contractility and enhances neuronal survivability in cells under pathological conditions, such as inflammations [53,54,55,56,57], whereas SOM changes motility and endometrial cell proliferation [58]. Moreover, it has recently been discovered that SOM influences contractile activity in inflamed uteri by increasing amplitude and decreasing frequency [59]. Under optimal conditions, NO is also known for its neuroprotective properties in both central and enteric nervous systems [32,60,61]. Although studies on immature pigs’ PCG have not found nitrergic perikarya [39], studies in rats and pigs indicate that the nitrergic nerve fibers supplying the uterine horn can derive from the mentioned ganglion [30,62]. The current results present a downregulation of cholinergic but upregulation of non-cholinergic, nNOS-immunoreactive neurons, which may be due to the increased importance of the nitrergic, sympathetic perikarya in a state of inflammation. This would be in line with the authors’ previous study on the sympathetic component of the PCG, in which a noradrenergic, nNOS-positive population noted a statistically significant increase [30].

Interestingly, the presented studies show the appearance of a minor cholinergic, GAL-expressing population and the emergence of a small population of GAL-positive, non-cholinergic neuronal cells in the *E*. *coli* treated group. It is generally known and accepted that GAL shows neuroprotective functions during brain injury and neurodegenerative diseases [63]. Alternatively, GAL may modulate the activity of other neurotransmitter systems, which, in turn, influence cholinergic transmission. Moreover, GAL has been proven to stimulate uterine contractility [64,65] and change the amplitude and frequency of uterine contractions [66]. Thus far, cholinergic, GAL-expressing neurons have been shown to exist in brain formations such as the basal forebrain and the hippocampus [67]. Research carried out on uterine innervation in immature pigs has shown no GAL-expressing populations [3] but uptake in GAL expression in response to axotomy was described earlier [39,40]. Endometritis-evoked emergence of VAChT+/GAL+ and VAChT-/GAL+ populations may suggest an increasing demand to upregulate this valuable neurotransmitter in order to benefit from its favorable properties. However, the rise in the non-cholinergic population is more significant, implying the greater importance of the non-cholinergic response of PCG neurons.

Regarding the earlier mentioned results of other authors, it has been shown that various treatments, either traumatic or non-traumatic, have detectable effects on cholinergic populations. The current results mainly showed an upregulation of ACh-expressing uterine neurons in response to endometritis, thus pointing to increased demand for ACh. It has been established that ACh exerts a vasodilatory effect in the uteri of guinea pigs and rats [68,69] and plays a role in increased contractility of the gilt uterus, supporting exudate removal [70,71]. Even though recent studies on the impact of Ach on contractility of the inflamed uterus confirmed its contraction-enhancing properties in groups without *E*. *coli* treatment, surprisingly, bacterial administration caused a decrease in the amplitude and an increase in the frequency of contractions by acting through muscarinic receptor 2 (MR2) and muscarinic receptor 3 (MR3). It may be speculated that this is due to changes in the expression of these receptors in response to inflammation, or it may result from the changes in their sensitivity [72,73]. It should be noted that the drop in contractility could also have resulted from the increased ACh effect and intensified production of myometrium relaxing substances, such as NO [72,74]. Moreover, parasympathetic nerve endings may be sources of NO in the inflamed uterus, as confirmed earlier in other inflamed organs [75]. In addition to having an effect on contractility, ACh influences the secretory activity of uteri in physiological conditions [76]. This fact may suggest that ACh plays a role in secretory mechanisms in the inflamed uteri as well.

It is worth mentioning that the number of neuronal populations expressing the examined neurotransmitters in cholinergic PCG perikarya is lower than in their noradrenergic equivalents. Moreover, the entire ACh-positive population is much lower in numbers than the population of the sympathetic counterparts [30]. Recent studies on the effect of partial hysterectomy on PCG neurons showed that, in immature pigs, cholinergic neurons supplying the right uterine horn were not present and did not appear after hysterectomy [40]. Since the results presented in this paper are the first to indicate the existence of such a type of perikarya in sexually mature gilts, it may be assumed that such populations appear after sexual maturation, albeit in small numbers. Additionally, the mentioned studies were performed on neurons innervating one of the horns, and data on how exactly this organ’s right and left part innervation is divided in PCG neurons are lacking [40]. It is known that the uterus exhibits physiological changes in response to the altered levels of sex hormones [19]. Such alterations, including estrogen levels during gilt’ maturation, may play a role in the differentiation of a minor number of neurons, possibly from a non-adrenergic, non-cholinergic (NANC) population [77], into a cholinergic phenotype, since it was proven that long-term E2 administration has an impact on chemical coding of ovarian perikarya in PCG [37]. Additionally, in rats, estrogen supplementation resulted in an increase in the diameter and density of the cholinergic nerve fibers in the uterus when applied during the infantile period [17].

Furthermore, the neuronal response to the bacterial administration of the cholinergic, neurotransmitter-positive uterine perikarya was less pronounced as a percentage, when compared with the dopamine beta-hydroxylase (DβH)-expressing population, as presented in a recent study by the authors [30]. However, the numbers of acetylcholine-positive, neurotransmitter-negative neurons showed a more marked increase, again implying a rising demand for ACh. In summary, such a situation may confirm that the sympathetic component of the PCG is more dominant in terms of uterine-horn innervation and it potentially plays a more pivotal role in the neurochemical expression of the described substances to pathological states, as shown in various different studies [29,30,40].

## 5. Conclusions

Intrauterine bacterial administration changes the neurochemical pattern organization of the cholinergic, uterus-innervating perikarya in the PCG, as demonstrated above. VAChT, SOM, nNOS, GAL, and VIP noted alterations in their expression, including AChE, emphasizing the significant adaptation capabilities of the uterus and the neurons supplying it. Additionally, the current results affirm the usability of pig models in studies on the effects of pathological states. The aim of science is to obtain novel data for further practical use and implementation. The collected results may find such an application and lead to the enhancement of reproductive indicators, increased profitability of animal production, and lower index of elimination from breeding by more efficient treatment of endometritis.

## Figures and Tables

**Table 1:** Antibodies used for immunostaining procedures.

Primary Antibodies				
**Antigen**	**Code**	**Host Species**	**Dilution**	**Supplier**
VAChT	V5387	rabbit	1:2000	Sigma-Aldrich, Saint Louis, MO, USA
VIP	ABS 023-02	mouse	1:1000	ThermoFisher Scientific Waltham, MA, USA
SOM	8330-0009	rat	1:60	Bio-Rad Laboratories, Watford, United Kingdom
GAL	T-5036	guinea-pig	1:2000	Peninsula, San Carlos, CA, USA
nNOS	N218	mouse	1:1000	Sigma-Aldrich, Saint Louis, MO, USA
**Secondary Antibodies**				
**Reagent**	**Code**		**Dilution**	**Supplier**
Alexa Fluor 546 nm goat anti-rabbit IgG	A21202		1:1000	ThermoFisher Scientific Waltham, MA, USA
Alexa Fluor 488 nm donkey anti-mouse IgG	A11010		1:1000	ThermoFisher Scientific Waltham, MA, USA
Alexa Fluor 488 nm goat anti-guinea pig IgG	A11073		1:1000	ThermoFisher Scientific Waltham, MA, USA
Alexa Fluor 488 nm donkey anti-rat IgG	A21208		1:1000	ThermoFisher Scientific Waltham, MA, USA

## Data Availability

All data generated or analyzed during this study are included in this published article.
